# Simultaneous quantification of trimethylamine N-oxide, trimethylamine, choline, betaine, creatinine, and propionyl-, acetyl-, and l-carnitine in clinical and food samples using HILIC-LC-MS

**DOI:** 10.1007/s00216-021-03509-y

**Published:** 2021-07-13

**Authors:** Mohammed E Hefni, Maria Bergström, Torbjörn Lennqvist, Cecilia Fagerström, Cornelia M Witthöft

**Affiliations:** 1 Department of Chemistry and Biomedical Sciences, Linnaeus University, 392 31 Kalmar, Sweden; 2 Food Industries Department, Faculty of Agriculture, Mansoura University, P.O. Box 46, Mansoura, 35516 Egypt; 3 Department of Health and Caring Sciences, Linnaeus University, 392 31 Kalmar, Sweden

**Keywords:** Methylamines, TMAO, TMA, Clinical samples, Food samples, LCMS

## Abstract

Trimethylamine-N-oxide (TMAO), a microbiome-derived metabolite from the metabolism of choline, betaine, and carnitines, is associated to adverse cardiovascular outcomes. A method suitable for routine quantification of TMAO and its precursors (trimethylamine (TMA), choline, betaine, creatinine, and propionyl-, acetyl-, and l-carnitine) in clinical and food samples has been developed based on LC-MS. TMA was successfully derivatized using iodoacetonitrile, and no cross-reactions with TMAO or the other methylamines were detected. Extraction from clinical samples (plasma and urine) was performed after protein precipitation using acetonitrile:methanol. For food samples (meatballs and eggs), water extraction was shown to be sufficient, but acid hydrolysis was required to release bound choline before extraction. Baseline separation of the methylamines was achieved using a neutral HILIC column and a mobile phase consisting of 25 mmol/L ammonium formate in water:ACN (30:70). Quantification was performed by MS using external calibration and isotopic labelled internal standards. The assay proved suitable for both clinical and food samples and was linear from ≈ 0.1 up to 200 μmol/L for all methylamines except for TMA and TMAO, which were linear up to 100 μmol/L. Recoveries were 91–107% in clinical samples and 76–98% in food samples. The interday (n=8, four duplicate analysis) CVs were below 9% for all metabolites in clinical and food samples. The method was applied successfully to determine the methylamine concentrations in plasma and urine from the subjects participating in an intervention trial (n=10) to determine the effect of animal food ingestion on methylamine concentrations.

**Graphical abstract:**

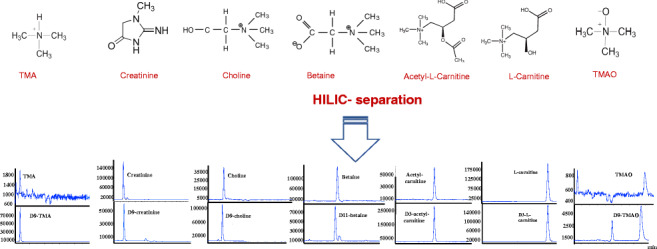

**Supplementary Information:**

The online version contains supplementary material available at 10.1007/s00216-021-03509-y.

## Introduction

The methylamines trimethylamine N-oxide (TMAO) and its precursors, trimethylamine (TMA), choline, betaine, and carnitines, have received increasing attention over the past decade owing to their potential link to adverse cardiovascular outcomes [1–4]. Choline, carnitines, betaine, and TMAO are metabolized into TMA by gut microbiota [5], which is rapidly oxidized by hepatic flavin monooxygenases into TMAO [5]. Hence, diet, gut microbiota composition, and liver flavin monooxygenase activity are the determining factors for TMAO in plasma. Animal-based foods (e.g., red meat, eggs, and dairy products) are rich in choline and different carnitine esters (free and esterified as phospholipids) [6, 7], and therefore are considered as potential sources of TMAO. This has led to an increased interest in studying the effect of a diet high in dietary methylamines on the formation of TMAO [8–11]. Such studies require an analytical method which allows simultaneous quantification of TMAO and related metabolites, not only in biological but also in food samples. To the best of our knowledge, no method for the simultaneous quantification of TMAO, TMA, choline, betaine, creatinine, and propionyl-, acetyl-, and l-carnitine in clinical and food samples is currently available.

While TMAO, choline, betaine, and carnitines can be measured directly using LC-MS/MS [12, 13], TMA, which does not allow fragmentation, needs to be derivatized. In addition, acetonitrile, which is frequently used in the mobile phase, forms adducts with ammonium ions (*m/z* = 59), which may interfere with the TMA signal at *m/z* = 60 [14]. A number of reagents have been developed to derivatize TMA (e.g., ethyl bromoacetate [11], tertbutyl bromoacetate [15], or iodo-acetonitrile/-acetamide [14]), but no studies have been reported focusing on the effectiveness of these derivatizing reagents.

Most of the present methods for the quantification of TMAO and its related metabolites have been restricted to small numbers of metabolites, focusing mainly on clinical samples. Available methods are based on nuclear magnetic resonance (NMR) spectroscopy [12, 13], liquid chromatography–tandem mass spectrometry (LC-MS/MS), or isotope dilution liquid chromatography-mass spectrometry [15–22]. A drawback of the mentioned methods is the narrow linearity range, which can be limiting when samples have a high variability in concentrations, particularly common in food samples. Although single quadrupole MS proved suitable for measuring some methylamine metabolites with a wider linearity range [14, 23], good chromatographic separation is required to overcome the lack of selectivity compared with LC-MS/MS. The high polarity of methylamines makes the use of reversed phase separation difficult due to the poor retention, which can be improved by using hydrophilic liquid interaction chromatography (HILIC). Therefore, the aim of the present study was to develop and internally validate a robust method using a HILIC separation mode and the widely available single quadrupole MS equipment for the simultaneous quantification of the most dominant methylamines in clinical and food samples (TMAO, TMA, betaine, choline, l-carnitine, acetyl-carnitine, and propionyl-carnitine). The study also includes an evaluation of different TMA derivatization methods. The analyte creatinine was included as it is commonly used to normalize data when analyzing urine. The usefulness of the method was demonstrated using a subsample of plasma and urine from subjects participating in an intervention trial to determine the effects of ingesting animal foods on methylamine concentrations in plasma and urine.

## Materials and methods

### Chemicals

TMAO, TMA betaine, choline, creatinine, l-carnitine, propionyl-carnitine, and acetyl-carnitine were purchased from Sigma-Aldrich (St. Louis, USA). Deuterated compounds (trimethylamine-d9, trimethylamine-N-oxide-d9, betaine-d11, creatinine-d3, l-carnitine-d3, acetyl-DL-carnitine-d3) were purchased from Cambridge Isotope Laboratories, Inc. (Andover, USA), and choline chloride-d9 from Sigma-Aldrich (St. Louis, USA). All other chemicals (iodoacetonitrile (IACN), ethyl bromoacetate (EBA), iodoacetamide (IAM), ammonium hydroxide, anhydrous sodium sulfate, methanol, and formic acid) were purchased from Sigma-Aldrich (St. Louis, USA), except acetonitrile (ACN), which was purchased from VWR International (Stockholm, Sweden). All chemicals were of p.a. grade, except acetonitrile and methanol, which were of HPLC grade. Water (MQ) was purified using a Milli-Q Water Purification System (Merck, Darmstadt, Germany).

Individual stock solutions of TMAO, TMA, betaine, choline, creatinine, l-carnitine, propionyl-carnitine, and acetyl-carnitine, as well as of the deuterated compounds, were prepared in MQ water with a concentration of 10 mmol/L and stored at −30 °C. Aqueous calibration solutions were prepared using serial dilution to contain 0.25, 0.5, 1, 2, 5, 10, 50, 100, and 200 μmol/L of the individual compounds in combination with a constant concentration of IS mixture (20 μmol/L of each deuterated compound). The internal standards were used to generate the calibration curve using the peak area ratio of the analyte to its deuterated compound. LOD and LOQ were estimated using the signal to noise (S/N) ratio of 3 and 10, respectively, for each compound.

### Derivatization of TMA

Three different derivatizing reagents (EBA, IAM, and IACN) were tested for derivatizing TMA.

For EBA, the derivatization reaction was carried out according to the method described by Cho et al. [11]. Briefly, 25 μL of TMA solution (10 mmol/L), 2 μL of concentrated NH_4_OH, and 30 μL of EBA solution (20 mg/mL in ACN) were mixed. Samples were incubated at room temperature for 45 min. To stop the reaction, the volume was brought up to 1 mL using a mixture of water:ACN:formic acid (1:1:0.0005) and transferred to HPLC vials. Different reaction conditions were tested (e.g., EBA concentration (30 vs 60 μL) and NH_4_OH volume (1, 2, 5, and 10 μL), incubation time (15, 30, and 45 min), and temperature (ambient temperature ~22 °C vs 6 °C))

For IAM, the derivatization reaction was carried out according to the method described by McEntyre et al. [14]. Briefly, 25 μL of TMA solution (10 mmol/L) was added to 960 μL of extraction solvent of water:methanol (9:1) and 15 μL of IAM (40 mmoL). Microcentrifuge tubes were half-filled with anhydrous sodium sulfate (~80 mg), vortexed for 5 min, and then centrifuged (3 min, 13,000g). Finally, sample supernatants were filtered through a syringe filter (0.45-μm pore size, polypropylene membrane, Agilent Technologies, Santa Clara, USA). Different reactions conditions were tested (e.g., different volumes from NH_4_OH (0, 2, 4, and 6 μL) and IAM (10 and 15 μL), extraction solvents (water, ethanol, mixture of ACN:MeOH 9:1), and reaction temperature (ambient temperature ~22 °C and 50 °C)).

For IACN, the derivatization reaction was carried out according to the method described by McEntyre et al. [14]. Briefly, 968 μL of the extraction solvent of acetonitrile:methanol (9:1) was transferred to a 1.5-mL microcentrifuge tube, and 25 μL of TMA solution, 2 μL of concentrated NH_4_OH, and 5 μL of IACN were added and vortexed. The tubes were half filled with anhydrous sodium sulfate (~80 mg), vortexed for 5 min, and centrifuged (3 min, 13,000g); this step was later omitted (as it did not affect the reaction). The extracts were filtered through a syringe filter (0.45-μm pore size). Different reaction conditions were tested, e.g., various volumes from NH_4_OH (0, 2, 4, and 6 μL) and from IACN (5, 10, 15, and 20 μL) and the need for anhydrous sodium sulfate.

### Sample preparation and extraction

#### Clinical samples (plasma and urine)

For method setup, plasma and urine samples were collected from a single donor 2 h after ingestion of two eggs. Aliquots of 250–500 μL were stored at −80°C. Samples were thawed at room temperature. Plasma samples were not diluted, but urine samples were diluted 1:5 in MQ water prior to analysis of TMAO and its related methylamine metabolites. A higher dilution (1:200) was applied for analysis of creatinine in urine.

For derivatization, 25 μL of sample (standard, plasma or diluted urine) was transferred to a 1.5-mL microcentrifuge tube and 10 μL IS (50 μmol/L), 5 μL IACN, and 2 μL NH_4_OH were added. The volume was adjusted to 1 mL with ACN:MeOH (9:1), and samples were vortexed (5 min) and then centrifuged (13,000g for 5 min). The supernatant was filtered (0.45-μm pore size) and transferred into HPLC vials.

As the urine pH varies (from 4 to 8) between individuals, the effects of different pH levels (2.5, 4, and 8) were tested and the results were compared with a native urine sample pH (6.5). The pH 2.5 was tested because it has been reported to improve the derivatization reaction [21]. Formic acid and ammonium hydroxide were used for pH adjustment. In most human intervention trials, urine is collected for 6 h. During this 6-h period, the samples are usually kept at room temperature, which might affect the stability of the targeted compounds. Therefore, a stability test was carried out by keeping a urine sample at room temperature for 6 h and the results were compared with the same freshly collected sample.

#### Food samples

Two food samples, meatballs and eggs, rich sources for choline, l-carnitine, creatinine, and to some extent betaine, were used for the method optimization. Meatballs (ingredients: beef and pork (73%), water, potato, potato flour, onion, iodine salt, potato fiber, dextrose, sugar, spices, and spice extracts) were purchased from a local supermarket (Kalmar, Sweden). The meatballs were microwaved for 2 min (according to the instructions on the label), freeze-dried (BenchTop Pro, VirTis, USA), milled using a coffee grinder (Melitta, Minden, Germany), and kept at −30 °C until analysis. Eggs from five different suppliers, including organic eggs, were purchased from a local supermarket and farm (Kalmar, Sweden). The eggs (one egg from each supplier) were boiled (11 min), freeze-dried, milled, and kept at −30 °C until analysis.

Two sample extraction procedures were tested for the extraction of the free dietary methylamines using pure water, based on the method suggested by Hefni et al. [24] and 50% methanol/water based on the method suggested by Bruce et al. [25]. To ensure complete extractability, samples were extracted sequentially three times and the extracts were analyzed separately. From this trial, it was determined that two extractions were enough (see “Results”), and the final protocol for extraction was as follows: 2 mL water was added to 100 mg freeze-dried food sample, vortexed (5 min), and centrifuged (5 min, 13,000 g). The resulting supernatant was transferred into a 10-mL polypropylene tube. The extraction was repeated by suspending the pellet in 2 mL water, vortexing (5 min), and centrifuging (5 min, 13,000 g). Both supernatants were collected in the same tube. An aliquot (25 μL) of the supernatant was transferred into a 1.5-mL microcentrifuge tube and derivatized, as described previously for the plasma and urine samples.

The extraction of total choline was carried out after acid hydrolysis [24]. Briefly, 100 mg of the freeze-dried food samples was homogenized into 5 mL 1M HCl and the mixture was incubated at 60°C overnight (18 h). Thereafter, samples were cooled to room temperature, neutralized (pH 5–6) using concentrated NH_4_OH, and the volume was adjusted to 10 mL using MQ-water. The sample hydrolysate was centrifuged (2600g for 15 min) and filtered (0.45-μm pore size), and 25 μL transferred into a 1.5-mL microcentrifuge tube and treated as previously described for the plasma and urine samples. To investigate the differences in methylamine content within the same provider, six eggs were boiled, prepared (as described above), and analyzed separately.

### Quantification of methylamines

Quantification was carried out by LC (Agilent 1200, Agilent Technologies, Santa Clara, USA) coupled to a single quadrupole mass spectrometer (Agilent 6130) equipped with an APCI-ES source. Methylamines were separated in a neutral HILIC column (ACE, 150 mm × 4.6 mm; particle size 3 μm) with a guard column (ACE, 3.0 mm × 4.6 mm; particle size 3 μm) containing the same packing material, using an isocratic mobile phase containing 25 mmol/L ammonium formate in water:ACN (30:70). The column was thermostatically controlled at 25 °C. The flow rate was set at 0.6 mL/min (different flow rates were tested at 0.4, 0.6, 0.8, and 1 mL/min), the injection volume was 10 μL, and the total run time was 14 min. Parameters were set as follows: drying gas flow 11.0 L/min, nebulizer pressure 55 psi, drying gas temperature 250 °C, and capillary voltage +3000 V (positive ionization). The electrospray ionization mass spectra (ESI-MS) recorded in the mass-to-charge ratio range from *m/z* 40–250 were collected during method optimization. For the final method, a selected ion monitoring (SIM) mode was used, and the following *m/z* values were used to detect the different protonated molecules [M+H]^+^ at *m/z* 118 for betaine, *m/z* 104 for choline, *m/z* 162 for carnitine, *m/z* 218 for propionyl-carnitine, *m/z* 204 for acetyl-carnitine, and *m/z* 114 for l-creatinine. With regard to TMAO, in addition to a protonated molecule [M+H]^+^ at *m/z* 76, a singly protonated dimer [2M + H]^+^ at *m/z* 151 was formed; and therefore, ions were collected at both *m/z* 76 and *m/z* 151. The *m/z* ratio for TMA differed depending on the derivatization agent as follows: *m/z* 146 for TMA-EBA, *m/z* 117 for TMA-IAM, and *m/z* 99 for TMA-IACN. For an internal standard, the following *m/z* ratios were used: *m/z* 85 for TMAO-d9, *m/z* 117 for creatinine-d3, *m/z* 165 for carnitine-d3, *m/z* 113 for choline-d9, *m/z* 129 for betaine-d11, *m/z* 207 for acetyl-DL-carnitine-d3, and *m/z* 108 for TMA-d9-IACN (Table 1). Mass accuracy was ± 0.5 units in all measurements. Since some of the compounds have close retention times and similar masses (e.g., choline vs creatinine or TMAO vs l-carnitine), the protonated molecules were collected in three separate windows to avoid any overlap (see Supplementary Information (ESM) Fig. S1).

**Table 1:** Calibration parameters, limit of detection and quantification, linear range, and molecular masses of methylamines

Methylamines (IS)	Mass (IS)	Calibration curve			LOD	LOQ	Linear range μmol/L
		Slope	Intercept	R^2^	μmol/L		
TMA(D9)	99(108)	0.0353	−0.0075	0.9991	0.049	0.165	0.165–100
TMAO(D9)*	76(85)	0.0309	0.0196	1.000	0.055	0.182	0.182–100
Choline(D9)	104(113)	0.0404	0.0673	0.9985	0.035	0.118	0.118–200
Creatinine(D3)	114(117)	0.0393	0.0504	0.9944	0.046	0.153	0.153–200
Betaine(D11)	118(129)	0.0393	0.0395	0.9993	0.052	0.173	0.173–200
Acetyl-carnitine(D3)	204(207)	0.0409	0.0468	0.9992	0.019	0.063	0.063–200
l-Carnitine(D3)	162(165)	0.0393	0.0504	0.9988	0.014	0.048	0.048–200
Propionyl-carnitine	217	0.0473	0.0535	0.9989	0.026	0.086	0.086–200

*TMA*, trimethylamine; *TMAO*, trimethylamine N-oxide; *IS*, internal standards; *R*^*2*^, correlation coefficient; *LOD*, limit of detection; *LOQ*, limit of quantification. Established by signal to noise (1:3 and 1:10) approach, respectively. *TMAO; in addition to a protonated molecule [M+H]^+^ at *m/z* 76, a singly protonated dimer [2M + H]^+^ at *m/z* 151 was formed, and therefore ions were collected at both *m/z* 76 and *m/z* 151

### Quality control of analytical method

To control for interday and intraday variation, plasma, urine, and lyophilized meatball samples were analyzed (four analysis occasions in duplicate). Recovery tests in clinical samples were carried out by spiking the plasma and urine samples with TMA, TMAO, betaine, choline, l-carnitine, creatinine, and acetyl carnitine. All metabolites were added to plasma samples at the levels of 5, 10, 20, 50, and 100 μmol/L. For urine, all metabolities were added at the levels of 5, 10, 20, and 50, except creatinine, which was added at the levels of 100, 200, 400, and 800 μmol/L, because of high creatinine concentration in the urine. Recovery tests in food samples were carried out by spiking with all compounds aiming at two different levels (50 and 100% of the inherent concentrations) (Table 3).

### Method application on clinical samples

The method was applied to a subsample of plasma and urine samples from subjects (n=10) participating in an intervention trial to determine the effect of animal foods on methylamine concentrations in plasma and urine. Blood samples were collected from January to May 2020 from ten healthy subjects, aged 57±6.7, after ingestion of a portion of 166±9 g of boiled eggs (corresponding to 3 hard-boiled eggs). Blood samples (3 mL) were withdrawn using an intravenous catheter from the arm at baseline and 0.5, 1, 2, 4, and 6 h after the test meal. Blood samples were centrifuged for plasma separation. Participants were asked to collect their baseline and post-dose urine over a 6-h period. Subsamples from collected plasma (250 μL) and urine (500 μL) were stored at −80 °C until analysis. The human trial was approved by the Regional Ethics Review Board in Linköping, Sweden (2019-04354).

### Calculations and statistics

The coefficient of variation (CV) for interday variation was calculated for clinical and food samples (n=8, four analysis occasions in duplicate). The recovery (R) was calculated according to the following equation: R=(C_found_−C_sample_)/C_added_, where C_found_ is the measured content in the spiked sample, C_sample_ is the measured concentration in the sample before spiking, and C_added_ is the added concentration. Data of methylamines in clinical and food samples was expressed as mean ± standard deviation (STD). One-factor analysis of variance for repeated measures (RM-ANOVA) with post-hoc Tukey comparison (GraphPad Prism 7.0 software) was used to analyze the differences in plasma and urine before and after egg ingestion. The level of significance was set to 0.05.

## Results

### *Derivatizing reagents*

IACN proved to be a suitable derivatizing reagent. It produces a cationic derivative which reacts with TMA to create a positively charged derivative that can be detected at *m/z* 99 by MS, according to the following reaction:

$$ \mathrm{N}\equiv \mathrm{C}-{\mathrm{CH}}_2\mathrm{I}+\mathrm{N}{\left({\mathrm{CH}}_3\right)}_3.\overset{{\mathrm{N}\mathrm{H}}_4\mathrm{OH}}{--\to}\mathrm{N}\equiv \mathrm{C}-{\mathrm{CH}}_2\hbox{--} {\mathrm{N}}^{+}{\left({\mathrm{CH}}_3\right)}_3+{\mathrm{I}}^{-} $$

The derivatization reaction only took place in the presence of NH_4_OH (ESM Fig. S2). However, the reaction was not sensitive to the concentrations of NH_4_OH or IACN, as varying these concentrations using 1–5 μL NH_4_OH and 5–10 μL IACN did not affect the yield determined by HILIC LC-MS (data not shown). The addition of a drying agent (anhydrous sodium sulfate) was not required (data not shown).

**Fig. 1:**
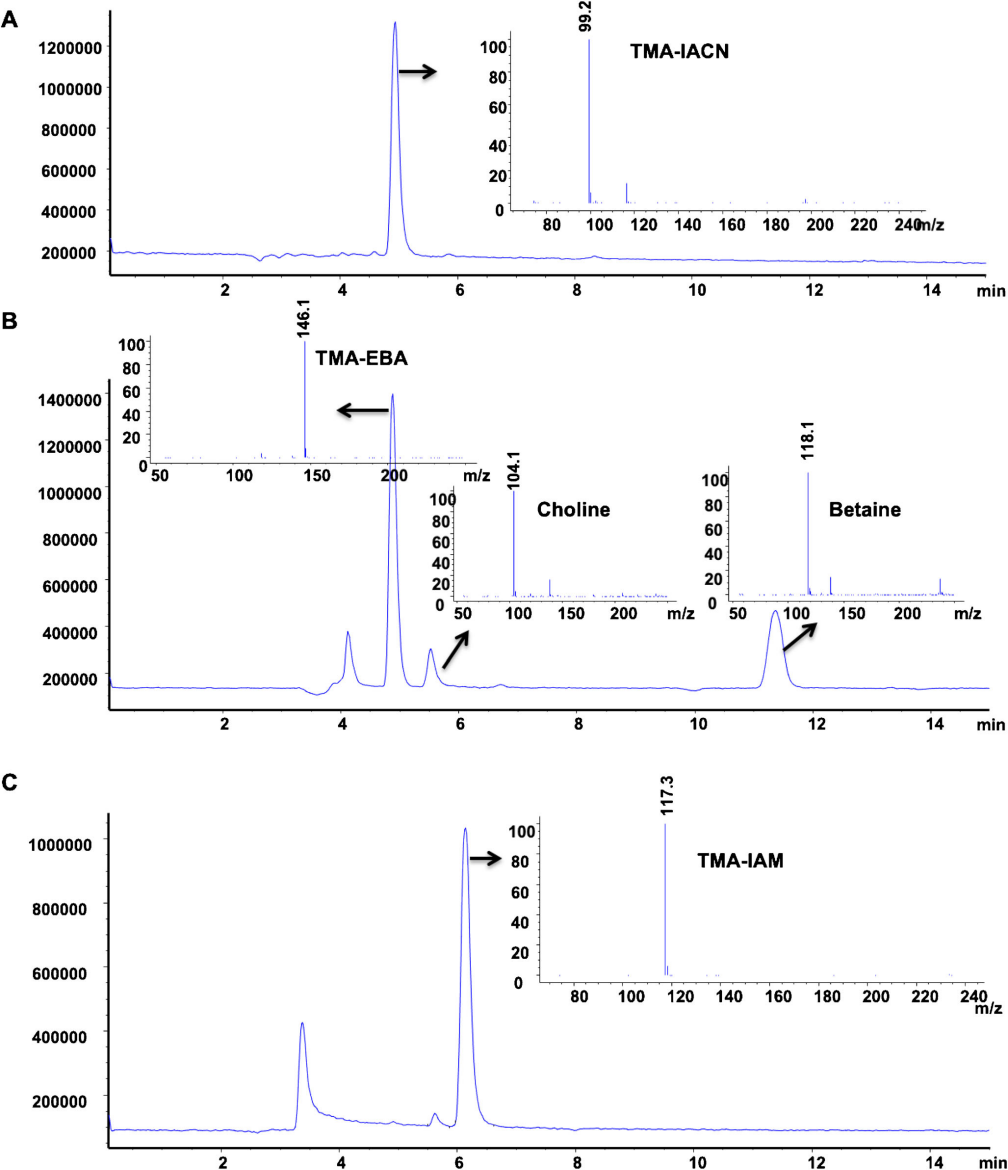
HILIC LC-MS separation of TMA (500 μmol/L) derivatized with **A** iodoacetonitril, **B** ethyl bromoacetate, and **C** iodoacetamide

The reaction of EBA with TMA was found to not only produce the TMA-ethyl-acetate derivative (targeted compound) but also choline and betaine (identified using the retention time and *m/z*) as by-products (Fig. 1B). In addition, the reaction was found to be sensitive to the concentration of NH_4_OH; the addition of 5 μL NH_4_OH was found to give the highest peak area. Other factors (e.g., EBA concentration, reaction time, and temperature) did not affect the yield or the reaction.

The reaction of IAM with TMA produced a cationic derivative (Fig 1C). However, the resulting TMA-IAM derivative has the same retention time as choline, and the peaks overlapped and could not be separated, even though different separation conditions were tried (data not shown).

### *Optimization of chromatographic conditions*

**Fig. 2:**
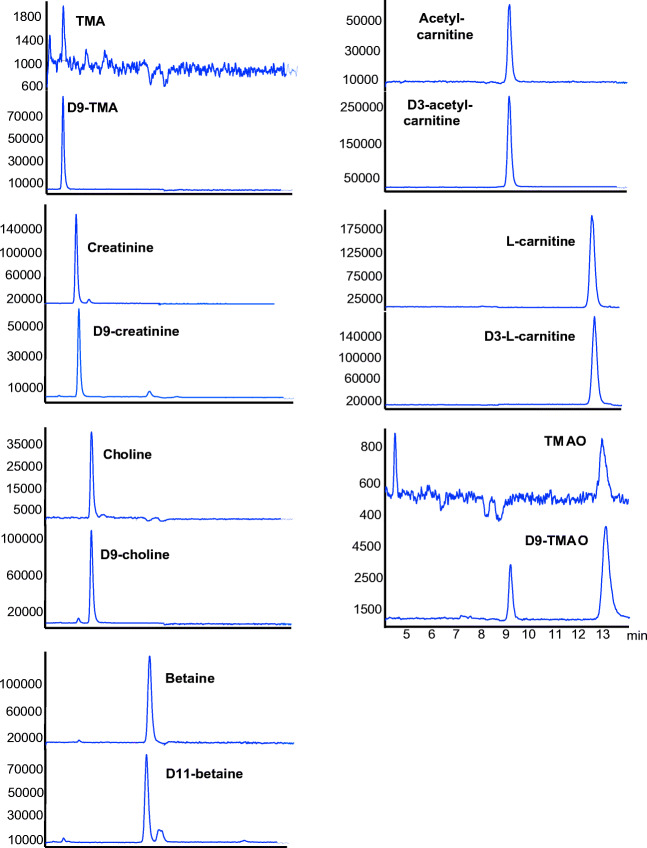
HILIC LC-MS separation of methylamines and their internal standards in plasma samples. The concentrations (μmol/L) were as follows: 0.9 for trimethylamine (TMA), 3.4 trimethylamine-N-oxide (TMAO), 10.9 for choline, 42.1 for betaine, 5.2 for acetyl-carnitine, 31.7 for l-carnitine, and 89.8 for creatinine. The concentration of the internal standards was 20 μmol/L

Isocratic elution with a mobile phase consisting of 25 mmol/L ammonium formate in water:ACN (30:70) showed a satisfying resolution of TMAO, TMA, choline, betaine, creatinine, propionyl-, acetyl-, and l-carnitine using a HILIC-N (neutral) column (Fig. 2). The addition of ammonium formate (25 mmol/L) to the mobile phase was required for both ionization and peak shape. Increasing the flow rate from 0.4 to 1 mL/min decreased the runtime from 25 to 11 min and did not affect the resolution, but the peak areas were not reproducible. The optimized flow rate was 0.6 mL/min, resulting in a total run time of 14 min and a good level of sensitivity and reproducibility (Table 4).

### *Extraction procedures for urine and food samples*

Neither differing pH values of urine nor 6 h-storage at room temperature (during simulation of sample collection on a study day) affected the concentration of compounds (ESM Table S1). The exception was that TMA did not react with IACN in urine at pH 2.5, probably because TMA is protonated (C_3_H_10_N^+^) and therefore less nucleophilic.

For food sample extraction, two solvents were tried, *pure* water and 50% MeOH in water; and as both showed similar results for all analyzed metabolites (Table 5), water was chosen as the standard method. It was found that two repeated extractions were enough to completely release the methylamines from the food samples. The first extraction released between 80 and 90% of the content. The second extraction released the remaining amounts, as no peaks were detected in the third extract (data not shown). This finding demonstrates the importance of repeating the extraction procedure for reliable quantification. Although acid hydrolysis is necessary to extract the total choline content, it proved to be unsuitable for l-carnitine esters and creatine. During acid hydrolysis, carnitine esters (acetyl-carnitine and propionyl-carnitine) were hydrolyzed to l-carnitine, which confirms earlier findings [7]. Furthermore, it has been reported that creatine was converted to creatinine [26] at an elevated temperature and/or at a low pH.

### *Quality control of the analytical method*

The calibration curves of all metabolites were linear up to 200 μmol/L, except TMAO and TMAO, which were linear up to 100 μmol/L (Table 1). The LOQ ranged from 0.048 for l-carnitine to 0.182 μmol/L for TMAO, which proved suitable for both food and clinical samples. The relative recovery of individual metabolites after standard addition (duplicate samples at each addition level) for both plasma (five levels, n=10) and urine (four levels, n=8) ranged from 91% (TMAO in plasma) to 107% (TMA in plasma) (Table 2). For all compounds, measured concentrations in the plasma samples after standard addition were linear and resulted in correlation coefficients R^2^ above 0.995 (ESM Fig. S3). For food samples, the relative recovery after standard addition to both egg and meatballs at two concentrations (duplicate samples at two addition levels) ranged from 76% (TMAO in eggs) to 98% (l-carnitine in meatballs) (Table 3). The interday variation (CV%, n=8, four analysis occasions in duplicate) for all metabolites in food and clinical samples (Table 4) was below 10.

**Table 2:** Concentrations of methylamines (μmol/L) in spiked and non-spiked plasma and urine clinical samples and the calculated average

Compound	Level of addition (μmol/L) (Recovery %±RPD)						Overall average recovery (%±RSTD)
	0	5	10	20	50	100	
Plasma							
TMA		6.2 (106.9±0.1)	11.6 (108.0±4.6)	21.9 (105.6±4.0)	55.6 (109.6±0.3)	106.2 (105.4±0.7)	107.1±1.5
TMAO	3.2	7.5 (86.0±5.2)	12.0 (87.3±3.5)	21.4 (90.8±1.1)	50.8 (95.0±2.0)	97.0 (93.7±0.8)	90.6±3.5
Choline	10.6	15.3 (95.8±7.7)	19.7 (91.7±0.2)	29.7 (95.7±1.4)	58.2 (95.3±1.0)	104.6 (94.1±0.3)	94.5±1.5
Betaine	41.9	46.4 (90.1±1.0)	51.1 (92.5±3.7)	61.2 (96.5±0.2)	93.5 (103.2±1.5)	132.3 (90.4±1.5)	94.5±4.9
Acetyl-carnitine	5.1	9.8 (95.4±0.6)	14.9 (98.5±1.7)	25.5 (102.1±0.4)	56.2 (102.3±0.6)	101.6 (96.5±1.8)	99.0±2.8
l-Carnitine	30.6	35.8 (105.6±6.8)	40.9 (103.3±3.0)	51.3 (103.5±3.6)	82.9 (104.7±0.2)	128.5 (97.9±0.7)	103.0±2.7
Creatinine	90.9	95.7 (96.2±24.9)	100.8 (99.4±12.5)	111.2 (101.4±7.8)	141.8 (101.9±5.6)	189.5 (98.6±2.6)	99.5±2.1
Urine*							
TMA	0.98	5.6 (93.1±1.4)	9.9 (94.3±3.7)	19.9 (97.5±1.5)	45.3 (91.2±0.6)		93.6±3.0
TMAO	9.8	14.4 (92.0±3.)	18.9 (92.8±1.0)	28.2 (95.7±3.4)	56.5 (93.5±0.9)		93.5±1.6
Choline	3.7	8.2 (93.4±0.2)	13.0 (97.9±0.3)	22.7 (97.8±0.7)	49.1 (94.1±1.6)		95.8±2.0
Betaine	5.7	10.8 (97.6±1.2)	15.9 (103.6±0.5)	26.2 (103.2±0.6)	54.7 (96.6±2.4)		100.3±3.6
Acetyl-carnitine	1.4	6.3 (94.8±0.8)	11.6 (100.7±1.2)	22.4 (101.7±0.3)	50.1 (98.5±1.6)		99.0±3.1
l-Carnitine	4.0	9.0 (101.5±2.0)	14.1 (100.9±0.4)	24.7 (104.2±0.4)	52.9 (98.9±0.4)		101.4±2.2
Creatinine**	862	976 (110.3±3.7)	1084 (106.7±2.8)	1330 (104.2±3.6	1706 (98.3±2.1)		104.9±5.1

*TMA*, trimethylamine; *TMAO*, trimethylamine N-oxide. Concentrations are means of duplicate samples (± relative percentage difference (*RPD*)). *RSTD*, relative standard deviation.

*Data was not corrected for dilution factor 5

**For creatinine, concentrations of 0, 100, 200, 400, and 800 μmol/L were instead added (described in “Quality control of analytical method” section)

Recovery (R)=(C_found_−C_sample_)/C_added_

**Table 3:** Concentrations of methylamines (μmol/L) in spiked and non-spiked food samples and the calculated average

Compound	C_sample_	C_added_	C_found_	Recovery (%)	Average recovery (%±RSD)
	(mg/100g FW)				
Meatball					
TMA	0	1.1	1.1	100.3±0.1	97.9±4.9
		2.8	2.6	95.4±6.8	
TMAO	0	1.8	1.2	83.3±5.6	82.4±3.6
		3.5	2.9	81.5±2.9	
Choline	31.5	19.8	53.1	109.6±3.3	101.6±11.5
		48.7	76.8	93.7±11.5	
Betaine	9.7	4.4	14.5	105.9±4.8	100.6±11.1
		10.9	20.2	95.5±15.5	
Total carnitine	11.3	6.1	17.9	107.2±0.1	97.8±12.4
		15.1	24.6	88.5±10.7	
Egg					
TMA	0	0.7	0.68	93.1±2.7	94.7±2.8
		1.3	1.27	96.4±2.4	
TMAO	0	0.9	0.7	71.8±3.2	75.8±7.1
		1.7	1.3	79.8±0.9	
Betaine	1.2	1.5	2.6	98.1±5.2	96.1±5.5
		2.6	3.5	93.9±6.8	
Choline	260.6	64.7	320.3	92.2±4.3	95.5±4.8
		116.5	375.9	98.9±2.2	
l-Carnitine	0	2.0	1.6	79.6±5.9	82.1±4.7
		3.6	3.1	84.7±1.9	
Creatinine	0	1.4	1.0	71.5±9.5	79.8±10.7
		2.5	2.2	88.1±7.7	

*TMA*, trimethylamine; *TMAO*, trimethylamine N-oxide. *C*_*found*_, mean content in the spiked sample; *C*_*sample*_, mean content in the sample without spiking; *C*_*added*_, content added to the sample. Contents are means of duplicate samples (± relative percentage difference). Further details are described in “Quality control of analytical method” section

Recovery (R)=(C_found_−C_sample_)/C_added_

**Table 4:** Interday variation (n=8) of the quantification of methylamines in the clinical and food samples

Matrix		TMA	TMAO	Choline	Betaine	Acetyl-carnitine	l-Carnitine	Creatinine
Plasma	Conc (μmol/L)	0.9	3.4	10.9	42.1	5.2	31.7	89.8
	CV (%)	7.6	8.5	6.3	2.9	2.1	3.5	1.0
Urine	Conc (μmol/L)	4.4	47.7	18.6	28.5	7.0	20.2	4339.8
	CV (%)	6.2	3.3	2.0	2.5	3.1	0.2	1.2
Meat	Content (mg/100g FW)			32.7	10.2		11.8	264.7
	CV (%)			5.04	3.9		3.63	2.5

Variation within a day (intraday) was lower than 5%. *TMA*, trimethylamine; *TMAO*, trimethylamine N-oxide. Both TMA and TMAO were not detected in the meatballs. Eggs were not included as in-house control sample to estimate interday variation since they contain only choline and to a lesser extent betaine. Plasma and urine were collected from one subject 2 h after ingestion of two eggs and used during method development

### *Method application*

The method was successfully applied to determine methylamines in animal foods (eggs and meatballs). Choline and, to a lesser extent, betaine were found in eggs (Table 5), whereas l-carnitine, choline, betaine, and creatinine were found in meatballs (Table 5). The average total choline content in boiled eggs obtained from different suppliers (n=5) varied from 216 to 288 mg/100 g fresh weight (data not shown). Analytical variation was less than 5% within the same batch of 6 eggs (data not shown).

**Table 5:** Methylamine content (mg/100 g FW) in food samples

Methylamines	Meat		Egg	
	Water (n=4)	Acid extract (n=12)	Water (n=10)	Acid extract (n=12)
Choline	1.4±0.0^a^	33±1.2^b^	0.3±0.1^a^	255±24^b^
Betaine	11±1.3	10±0.3	0.8±0.1	0.9±2.8
Creatinine	31±5.3	270±8.7	nd	nd
l-Carnitine	11±1.0	12±0.3 ^c^	nd	nd
Acetyl- carnitine	2.4±0.3	nd	nd	nd

*nd*, not detected. Methylamines TMA and TMAO were not detected

^a^Free choline

^b^Total choline

^c^Total carnitine

In clinical samples of 10 participants, the metabolites TMAO, TMA, choline, betaine, creatinine, and acetyl- and l-carnitine were determined simultaneously before and after ingestion of three hard-boiled eggs (Table 6). After ingestion, the concentrations of choline and betaine in plasma at C_max_ increased by 85% and 50%, respectively (p < 0.01) (Table 6), while the concentrations of TMAO and other metabolites remained the same. In postprandial urine, no significant changes in metabolite concentrations (normalized for creatinine concentration) were detected.

**Table 6:** Methylamines in plasma and urine after the ingestion of eggs (n=10 subjects ± SD)

Metabolite	TMA	TMAO	Choline	Betaine	Acetyl-carnitine	l-Carnitine	Creatinine
Oral dose (mg/portion)^a^	nd	nd	357±16.5	1.7±0.1	nd	nd	nd
Plasma (μmol/L)							
C_0_^b^	0.48±0.09	4.1±1.8	6.3±1.2	24.2±5.2	5.6±1.9	25.6±2.3	70.8±11.7
C_max_^c^	0.51±0.13	4.1±1.8	11.7±3.4**	36.4±9.7**	6.5±1.8	26.5±4.6	70.9±14.2
C_360_^d^	0.49±0.12	2.8±0.9	8.7±2.7	33.0±5.9	6.5±1.8	25.1±3.5	62.2±11.9
Urine^f^ (mmol/mol creatinine)							
C_pre-dose_^b^	1.4±1.1	64.4±24.1	0.8±0.6	7.0±8.5	0.38±0.8	2.11±1.11	na
C_post-dose_^e^	2.4±1.1	62.9±22.3	1.9±1.8	11.2±8.3	0.33±0.9	1.09±1.67	na

^a^Mean content of methylamines in three hard-boiled eggs (166 ±9)

^b^Concentration before egg ingestion

^c^Maximum plasma concentrations: TMAO at 0 min; creatinine at 30 min, choline at 120 min; betaine. TMA, l-carnitine at 240 min; acetyl-carnitine at 360 min

^d^Plasma concentration at 360 min after egg ingestion (last sampling)

^e^Urine concentration after egg ingestion and up to 360 min

*nd* not detected

^f^Data for urine were normalized for creatinine concentrations. Creatinine concentrations (n=10) range from 1506 to 16,834 μmol for pre-dose urine and 1133 to 4949 μmol for post-dose urine (collected until 360 min)

**Significant differences (**p<0.01). *na*, not applicable

## Discussion

Due to the high cost of tandem mass spectrometry instruments, we aimed to examine whether single quadrupole mass spectrometry (LC-MS) can be used for the valid quantification of TMAO and its precursors (TMA, choline, betaine, creatinine, and propionyl-, acetyl-, and l-carnitine). Single LC-MS is limited—as compared to tandem MS—by a lower selectivity, as it measures only the molecular ion using selected ion monitoring (SIM). As a consequence, compounds with the same mass have to be chromatographically separated in order to be quantified. We therefore focused, initially, on a chromatographic method allowing good retention and complete separation of all metabolites. Due to the high polarity of the analyzed metabolites, a HILIC separation mode was implemented. There are several choices of commercially available HILIC columns with acidic, basic, or neutral characters. All three HILIC phases were tested and shown to provide different retention behaviors (data not shown). Good retention and a satisfying resolution were achieved using a neutral HILIC column (Fig. 2).

The derivatization procedure was optimized in order to achieve accurate quantification of TMA in clinical and food samples. With the exception of TMA, all other metabolites can be measured directly by LCMS. The low molecular mass of TMA (MW 59Da) makes direct quantification difficult due to background noise. The evaluation of three different derivatizing agents (EBA, IAM, and IACN) showed that the TMA can be easily measured after a simple and robust derivatization procedure using IACN. However, IACN failed to react with TMA in the absence of NH_4_OH (ESM Fig. S1), which may explain the very low response reported in an earlier study [14]. NH_4_OH has pKa of 8.86, which is close to that of TMA (9.57) and catalyzes the reaction. Additionally, at an alkaline pH, TMAO remains uncharged (pKa of TMAO is 4.7) [27–29], protecting TMAO from cross-reaction with IACN, as previously reported [14].

TMA was also alkylated with EBA producing a cationic quaternary ammonium ethyl acetate ester (*m/z* 146) from releasing of the bromine group. ESI-MS obtained by the reaction of TMA with EBA showed the formation of betaine and choline (which could interfere with native choline and betaine in the samples), therefore lowering the accuracy and precision of the method. Iodoacetamide also reacted with TMA. The derivative formed by the reaction of TMA with IAM has a molecular mass of 117 Da, which is very close to that of D3 creatinine (MW 117.2 Da), and only differing one mass unit from the betaine molecular ion at *m/z* 118 Da. In addition, the IAM-TMA derivative coeluting with choline and the quick recrystallization process of IAM solutions make the compound difficult to handle.

IACN was found to be the most applicable derivatizing reagent for various reasons: Firstly, iodide is a much better leaving group than bromide, meaning the reaction proceeds more easily and quickly (2–5 min compared with 45 min when using EBA). Secondly, IACN did not react or interfere with the quantification of the other metabolites. Thirdly, water from the samples (plasma, urine, or food extract) did not affect the reaction of IACN and TMA, meaning the addition of a drying agent (anhydrous sodium sulfate) was not required, thereby simplifying the extraction procedure.

The limit of quantification for all methylamines in the current study is similar to those obtained by tandem MS [11, 15, 21] and ranged from 0.048 μmol/L for l-carnitine to 0.182 μmol/L for TMAO, which is much lower than that for NMR methodology [12, 13]. This allows accurate quantification of physiological concentrations of TMA and TMAO in samples from clinical trials as well as food samples. Another advantage of the current single MS method is that it had a wider linearity range (up to 100 μmol/L for TMA and TMAO and 200 μmol/L for the other metabolites). The use of labelled internal standard ensured the precision of the analysis by correcting for variation from sample preparation as well as instrumental detection.

### *Method application*

The food samples (eggs and meatballs) were chosen because they are rich sources of choline, l-carnitine, creatinine, and, to some extent, betaine. In addition, there is an increased interest in studying the association between consumption of a diet high in dietary methylamines, gut microbiota, and the formation of TMAO [8, 11]. The average total choline content quantified in boiled eggs (216–288 mg/100g FW) was similar to that reported in the USDA database [6], confirming that eggs are a rich source of choline. In the USA [30], as well as Europe [31], eggs are reported to be the major source of choline in the diet. To our knowledge, no data on choline is available in European food composition databases. Therefore, resourses like the USDA database have to be used for calculating dietary choline intake [31], although this could lead to inaccuracies due to a lack of data on traditional foods and variation due to genus and species. Comparison of our own findings regarding methylamine content in meatballs with literature was difficult as the meatballs were a complex mix of pork, beef (unknown which cuts), and other ingredients. In earlier studies [6, 7, 32], the content of methylamine was found to depend not only on the meat type but also the meat cuts.

The method was successfully applied to a subsample of clinical samples. Quantified concentrations and the nature of detected methylamines are consistent with data reported in the Human Metabolome Database (www.hmdb.ca) and other studies [8, 11]. After the ingestion of eggs, only betaine and choline increased in plasma, whereas TMAO and other metabolites remained unchanged, supporting previous data [33]. Concentrations of methylamines, normalized for creatinine, did not alter between predose and postdose urine, but the method proved suitable to quantify physiologic concentrations of methylamines and creatinine in urine, as previously reported [11, 15, 21].

## Conclusions

A robust single quadrupole mass spectrometry (LC-MS) method suitable for quantifying TMA, TMAO, betaine, choline, l-carnitine, acetyl-carnitine, propionyl-carnitine, and creatinine was developed for both clinical and food samples. The method involves a robust and fast derivatization step and was successfully applied to determine methylamines in animal foods (meatballs and eggs) and clinical samples (plasma and urine).

## Supplementary Information

ESM 1 (DOCX 2587 kb)

## Data Availability

Not applicable
