# Supplementary material for: Simultaneous quantification of trimethylamine N-oxide, trimethylamine, choline, betaine, creatinine, and propionyl-, acetyl-, and l-carnitine in clinical and food samples using HILIC-LC-MS
Source: Anal Bioanal Chem. 2021 Jul 13;413(21):5349–60. doi: 10.1007/s00216-021-03509-y (PMC8405501; doi:10.1007/s00216-021-03509-y)
Supplement: Supplementary file 1 — (DOCX 2587 kb) [file 216_2021_3509_MOESM1_ESM.docx]

Table S1. Concentrations of methylamines (µmol/L) in urine – effect of storage temperature and pH

| Compound  (µmol/L) | Urine stored at room temperature before freezing |  | Urine immediately frozen after collection | | | |
| --- | --- | --- | --- | --- | --- | --- |
|  | pH 6.46 (native) |  | pH 6.46 (native) | pH 2.5 | pH 4 | pH 8 |
| TMA | 4.3 |  | 4.1 | 0.0 | 4.1 | 4.2 |
| TMAO | 45.1 |  | 46.9 | 45.3 | 44.7 | 45.3 |
| Choline | 18.6 |  | 19.1 | 18.4 | 18.9 | 18.4 |
| Betaine | 28.8 |  | 29.2 | 26.7 | 27.8 | 27.5 |
| Acetyl-carnitine | 6.5 |  | 7.0 | 6.4 | 6.6 | 6.3 |
| L-carnitine | 19.6 |  | 20.3 | 18.3 | 17.2 | 17.6 |
| Creatinine | 4233.5 |  | 4418.3 | 4003.0 | 4008.3 | 4075.9 |

Formic acid and ammonium hydroxide were used for pH adjustment. TMA did not react with IACN at pH 2.5. To simulate real sampling conditions during a study day, urine samples were stored at ambient temperature for 6 h prior to storage (-80 °C) before analysis.


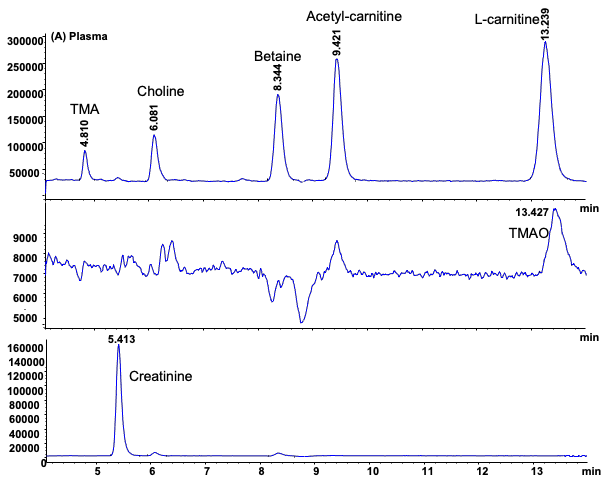


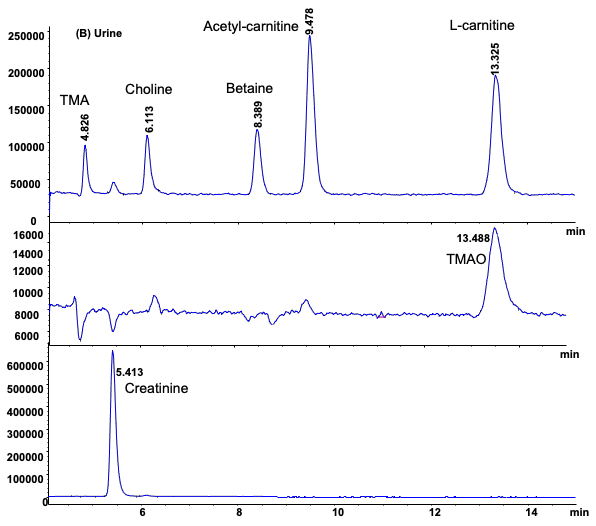


Fig. S1 Total ion chromatogram in SIM mode of methyl amines and precursors and their internal standards in (A) a plasma (undiluted) and (B) a urine sample (dilution 1:5). Details on sample preparation are presesented in the chapter Sample preparation and extraction, Clinical samples (plasma and urine).

**min**

**0**

**2**

**4**

**6**

**8**

**10**

**12**

**14**

**0**

**50000**

**10000**

**150000**

**200000**

**250000**

**300000**

**350000**

**400000**

**5µL NH_4_OH**

**2µL NH_4_OH**

**1µL NH_4_OH**

**0µL NH_4_OH**

Fig. S2. HILIC LC-MS separation of TMA standard (400 μmol/L) after derivatization by IACN with different volumes of NH_4_OH (see materials and methods section for derivatization of TMA and chromatography conditions)

Fig. S3. Correlation coefficients of added trimethylamine (TMA). trimethylamine-N-oxide (TMAO). choline. betaine. acetyl-carnitine. l-carnitine and creatinine to a plasma sample (see Quality control of analytical method in Materials and methods section)
